# Comparison of BEAM vs. LEAM regimen in autologous transplant for lymphoma at AIIMS

**DOI:** 10.1186/2193-1801-2-489

**Published:** 2013-09-26

**Authors:** Atul Sharma, Smita Kayal, Sobuhi Iqbal, Prabhat Singh Malik, Vinod Raina

**Affiliations:** Department of Medical Oncology, Dr. B. R. A. Institute Rotary Cancer Hospital, All India Institute of Medical Sciences, New Delhi, India

**Keywords:** Lymphoma, BEAM, LEAM

## Abstract

BEAM (BCNU, etoposide, cytrabine, melphalan) is the most widely used high dose chemotherapy regimen for autologous transplant in lymphoid malignancies. We report our early experience with an alternative regimen LEAM where BCNU was replaced with the oral analogue CCNU (lomustine) to tide over the non-availability of BCNU. Fifty one patients of relapsed or refractory lymphoma who received BEAM (n= 34) and LEAM (n= 17) from September 2001 to February 2012 were analyzed. From October 2009 onwards LEAM was used as the conditioning regimen instead of conventional BEAM. Patients in the LEAM group had more chemorefractory disease (35% vs 9%, p = 0.045) and high risk comorbidity score (24% vs 0%, p = 0.019). Grade 3 and 4 oral mucositis (67.6% vs. 64.7%, p = 0.834) and diarrhea (47% vs. 41.1%, p = 0.691) were similar. No difference was noted between the two groups in terms of engraftment, documented infections, antibiotic use, cumulative toxicity risk, length of hospital stay and 100 day transplant related mortality. The estimated 2 year overall survival (61.7% vs. 62.7%, p = 0.928) and event free survival (44.6% vs. 41.1%, p = 0.510) of the regimens BEAM and LEAM respectively were comparable. Thus LEAM appeared equivalent to BEAM in terms of toxicity and efficacy and can be used as an alternative to BEAM.

## Background

High dose chemotherapy (HDC) and autologous stem cell transplantation (ASCT) is an established treatment procedure and offers a chance for long-term disease control in many patients with relapsed or refractory Hodgkin’s lymphoma (HL) and Non Hodgkin’s lymphoma (NHL) (Appelbaum 2008). Several HDC regimens with different drug combinations have been in use, however, no randomized data is available to demonstrate superiority of any one regimen and retrospective comparative reports are sparse (Jo et al. 2008; Puig et al. 2006; Salar et al. 2001; Wang et al. 2004; Zaucha et al. 2008). Given the limited morbidity and comparable efficacy of the BEAM (BCNU/Carmustine, etoposide, cytrabine, melphalan) protocol, which was designed to avoid the toxicities of Cyclophosphamide and total body irradiation, it has become the most widely used conditioning regimen for lymphoma transplants (Linch et al. 1997; Mills et al. 1995). Lomustine (CCNU) based regimens have been used for high dose chemotherapy conditioning (Perz et al. 2007; Ramzi et al. 2012), mainly to overcome the increased pulmonary toxicity associated with BCNU (Stuart et al. 2001), but few if any comparative studies exist (Kumar et al. 2009). Recently, there was a shortage of BCNU globally and to overcome the situation we instead used a modified regimen LEAM, where BCNU was replaced with CCNU. In this study we report the results of this alternative drug schedule and compare it with the BEAM protocol, focusing on early toxicities, infectious complications and transplant-related mortality (TRM).

## Patients and methods

### Patient selection

The study group consisted of adult patients with relapsed or refractory lymphoma (NHL or HL) who were treated with high dose chemotherapy (either BEAM or LEAM) followed by autologous stem cell transplantation in the Department of Medical Oncology at the Institute Rotary Cancer Hospital, All India Institute of Medical Sciences (New Delhi, India) between September 2001 and February 2012. In all, a total of 51 patients were analyzed, 34 patients received BEAM and 17 patients received LEAM.

### Transplant protocol

Prior to transplant, patients were assessed for their clinical and treatment history, previous chemotherapy received including salvage therapy, pretransplant disease status, ECOG performance status, laboratory parameters and organ functions. Scores as per the hematopoietic cell transplant comorbidity index (HCT – CI) system (Sorror et al. 2005) for comorbidity risk assessment were calculated for each patient from clinical records and documented pretransplant laboratory evaluation and a score of 0 was assigned for absent information on a particular comorbidity. Growth factor (G-CSF, granulocyte colony stimulating factor) mobilized peripheral blood, which was harvested by leukapheresis, was the source of stem cell rescue for all patients. High dose chemotherapy regimen administered was BEAM in 34 patients and LEAM in 17 patients. Anti-emetic support included aprepitant, ondansetron, and dexamethsone with both the regimens. BEAM consisted of BCNU (carmustine) 300 mg/m2 (total dose) i.v. on day -6, etoposide 800 mg/m2 (total dose) i.v. divided over 4 days from days -5 to -2, ara-C (cytarabine) 1600 mg/m2 (total dose) i.v. twice daily divided over 4 days from days -5 to -2, and melphalan 140 mg/m2 (total dose) i.v. on day -1. LEAM consisted of CCNU (Lomustine) 200 mg/m2 p.o. on day -6, etoposide 800 mg/m2 (total dose) i.v. divided over 4 days from days -5 to -2, ara-C (cytarabine) 1600 mg/m2 (total dose) i.v. twice daily divided over 4 days from days -5 to -2, and melphalan 140 mg/m2 (total dose) i.v. on day -1. We cryopreserved stem cells in 18 patients and in 33 patients stem cells were stored at 4°C. Following infusion of the stem cell product (on day 0) after the end of conditioning, all patients were observed for toxicities and febrile complications which were managed as per standard guidelines and institutional protocol. All patients received growth factor support starting on day 1 till the time of engraftment. Platelet and blood transfusion was given as required during the course. All patients were treated as in-patients and remained hospitalized until engraftment and till the time deemed clinically suitable for discharge. Follow up information was collected by review of out-patient follow up records and patients were contacted through phone calls and letters.

### Outcome evaluation and study definitions

For the purpose of analysis standard remission criteria were applied to assess disease status following salvage chemotherapy, prior to HDC and transplant, and response beyond 30 days of transplant. Achievement of at least a PR after salvage therapy was regarded as chemosensitive disease. Patients with progressive or refractory disease prior to transplant were defined to have chemoresistant disease. Neutrophil engraftment was defined as first of three consecutive days with achievement of absolute neutrophil count of ≥ 500 /cm^3^ and no subsequent decline. Platelet engraftment was defined as first of three consecutive values of platelet count ≥ 20,000 /cm^3^ with transfusion independence Standard definition for febrile neutropenia was used and febrile episodes were classified according to IHS (Immunocompromised Host Society) consensus conference and the ESCMID (European Society of Clinical Microbiology and Infectious Diseases) guidelines into clinically documented infections (CDI), microbiologically documented infections (MDI) and fever of unknown origin (FUO) (Hughes et al. 1992; Pizzo et al. 1990). Regimen related organ toxicities, evaluated in the first 100 days, were graded using the Seattle criteria (Bearman et al. 1988). The maximum toxicity score was the highest score reached in any single organ system. Cumulative toxicity score was the sum of the highest score observed in each organ at any time. Length of hospital stay (LOS) was defined as the time from the day of infusion of stem cell product to the day of hospital discharge. Transplant related mortality (TRM) was taken as death from any cause other than disease relapse or progression occurring within the first 100 days after ASCT. Relapse or progression was defined as worsening in the disease status from that at the time of transplant or the start of a new definitive therapy at any time after transplant. Overall survival (OS) was measured from date of transplant to death from any cause. Event free survival (EFS) was defined from the day of transplant until death from any cause, relapse or progression.

### Statistical analysis

For evaluation and comparison of the early post transplant outcomes between the two groups of patients, we used the Fisher’s exact test for categorical variables and the Mann Whitney U test/ independent sample t test for continuous variables. OS and EFS were estimated using the Kaplan and Meier method. Log-rank test was used to examine differences in survival curves. Median follow up time was calculated using the Kaplan Meier method for potential follow up. Data were censored for survival analysis on 30^th^ June 2012. SPSS v 16.0 was used for analysis.

## Results

### Patient characteristics

Out of a total of 51 patients included in the study, 26 patients had NHL and 25 patients had HL. The median age of the study group was 33 years (range, 17 – 63 years) and the male to female ratio was 3.6:1. The median time from diagnosis to transplant for the whole cohort was 22.2 months (range, 1.9 – 144.1 months). The patient characteristics at transplant are outlined in Table 1 according to the high dose regimen. As the analysis was done retrospectively some differences were noted between the two groups. Considerably more patients in the LEAM group than the BEAM group had chemorefractory disease (35% vs 9% respectively, p = 0.045) and a high risk comorbidity score (24% vs 0% respectively, p = 0.019). The remaining pre-transplant features were comparable between the two groups.

**Table 1:** **Patient characteristic pre-transplant**

High dose chemotherapy regimen					
	BEAM (n = 34)		LEAM (n= 17)		
	N	%	N	%	p
Median age (range)	37 (17 – 63)		26 (18–59)		0.070
Sex					0.472
Male	28	82	12	71	
Female	6	18	5	29	
Diagnosis					0.771
NHL	18	53	8	47	
HL	16	47	9	53	
Number of prior chemotherapy lines					0.638
1	3	9	3	18	
2	14	41	7	41	
≥ 3	17	50	7	41	
Median time Dx to transplant (months)	24.3 (1.9 – 128.7)		17.3 (2.0 – 144.1)		0.187
Pre transplant disease status					0.074
CR	13	38	5	30	
PR	18	53	6	35	
Progressive/ refractory disease	3	9	6	35	
Chemosensitivity					0.045
Chemosensitive disease	31	91	11	65	
Chemorefractory disease	3	9	6	35	
ECOG PS					0.650
0 – 1	28	85	16	94	
≥ 2	5	15	1	6	
HCT-CI					0.019
Low risk (0)	19	56	8	47	
Intermediate risk (1 – 2)	15	44	5	29	
High risk (≥ 3)	0	-	4	24	
Median CD34 cell dose	2.3 x 10^6^/kg		2.4 x 10^6^/kg		0.536

*Abbreviations*: *NHL*, Non Hodgkin’s lymphoma; *HL*, Hodgkin’s lymphoma; *Dx*, Diagnosis; *CR*, Complete remission; *PR*, Partial remission; *ECOG PS*, Eastern Cooperative Oncology Group Performance Status; *HCT-CI*, Hematopoietic Cell Transplant Co morbidity Index.

### Peri-transplant outcomes

We observed no significant difference in the peri-transplant outcomes in terms of engraftment, febrile complications, regimen related organ toxicities, intravenous antibiotic use, duration of hospitalization and 100 days TRM between the two high dose regimens studied. The early post transplant outcomes are summarized in Table 2 in relation to the study subgroups.

**Table 2:** **Peritransplant outcomes**

High dose chemotherapy regimen					
	BEAM (n = 34)		LEAM (n= 17)		
	N	%	N	%	p
Engraftment (days)*					
Neutrophil engraftment	12 (8 – 41)		15 (10 – 25)		0.090
Platelet engraftment	18.5 (10 – 37)		22 (12 – 35)		0.199
Febrile neutropenia					0.806
FUO (Fever of unknown origin)	9	26.5	6	35.3	
MDI (microbiologically documented infection)	14	41.2	6	35.3	
CDI (Clinically documented infection)	11	32.3	5	29.4	
Antibiotic days *	13 (5 – 57)		18 (12 – 27)		0.185
Antibiotic number^†^	4.9 ± 1.6		5.7 ± 2.3		0.157
Regimen related Organ toxicities					
Oral mucositis (Grade 3/4)	23	68	11	65	0.834
Diarrhea (Grade 3/4)	16	47	7	41	0.691
Pulmonary dysfunction (Grade 3/4)	3	9	1	6	1.00
Cumulative toxicity risk score (Seattle criteria)					
Low risk (0 – 4)	22	65	13	76	0.448
Intermediate risk (5 – 8)	5	15	3	18	
High risk (≥ 9)	7	20	1	6	
Length of hospital stay (days)*‡	20.5 (12 – 80)		25 (15 – 31)		0.414
100 days TRM					
Yes (died)	6	18	2	12	0.703
No (Alive)	28	82	15	88	

* median values with range.

† mean number of antibiotics used per patient if a given antibiotic was used intravenously for more than 48 hours.

‡ from day of infusion of stem cell graft to day of hospital discharge.

### Engraftment

The median number of progenitor CD34+ cells harvested were comparable in the two groups (p = 0.536) and the time to neutrophil engraftment was similar (12 and 15 days for BEAM and LEAM respectively, p = 0.090). The median time to platelet recovery was also comparable (18.5 and 22 days for BEAM and LEAM respectively, p = 0.199).

### Infectious complications

All patients developed neutropenic fever at a median of 3 days after the infusion of stem cell graft. The febrile episodes were clinically documented in 29% (n = 15) patients, microbiologically documented in 39% (n = 20) patients and were without a defined focus in 32% (n = 16) patients. There was no difference in the occurrence of complicated infections between the two HDC subgroups. The number of broad spectrum antibiotics used and median duration of antibiotic use were also comparable (p = 0.157 and 0.185 respectively) as shown in Table 2.

### Regimen related toxicities

The most common grade 3 or 4 organ toxicity was mucositis and was observed in 67% patients in all, followed by diarrhea in 45% patients. Distribution of grade 3 or 4 mucositis and diarrhea in the two groups were similar. Grade 4 pulmonary toxicity was observed in 4 patients that was similarly distributed between the two groups. Grade 3 or 4 hepatic toxicity was seen in 3 patients, all in the BEAM subgroup. No grade 3 or 4 renal toxicity was noted, however, grade 1 / 2 renal dysfunction occurred in 16 patients (31%) and was similarly distributed in both the groups (p = 0.753). Cardiac toxicity (grade 1) was seen in only 1 patient with NHL in the BEAM arm. Grade 3 or 4 CNS (Central nervous system) toxicity was noted in 1 patient in the BEAM group. Four patients had hemorrhagic cystitis, 3 in the BEAM and 1 in the LEAM arm. However, the cumulative toxicity risk scores, calculated according to the Seattle criteria as described, were equitably distributed between the two study groups with comparable numbers in the low, intermediate and high risk categories as shown in Table 2. The median duration of hospitalization at 20 days and 25 days for the BEAM and the LEAM arms respectively was also comparable (p = 0.414).

### Transplant related mortality

TRM at 100 days occurred in 8 patients (15.6%), 6 patients (18%) died in the BEAM group and 2 (12%) patients died in the LEAM group (p = 0.703). Majority of the patients (n = 7) died prior to neutrophil engraftment and the causes for death were sepsis in 2 patients, multiorgan dysfunction (primarily respiratory) in 4 patients and multifactorial (infections plus organ toxicities) in 1 patient. One patient died on day 41 of transplant due to multiorgan failure and sepsis. All four patients who developed grade 4 pulmonary toxicity died.

### Survival results

**Figure 1:**
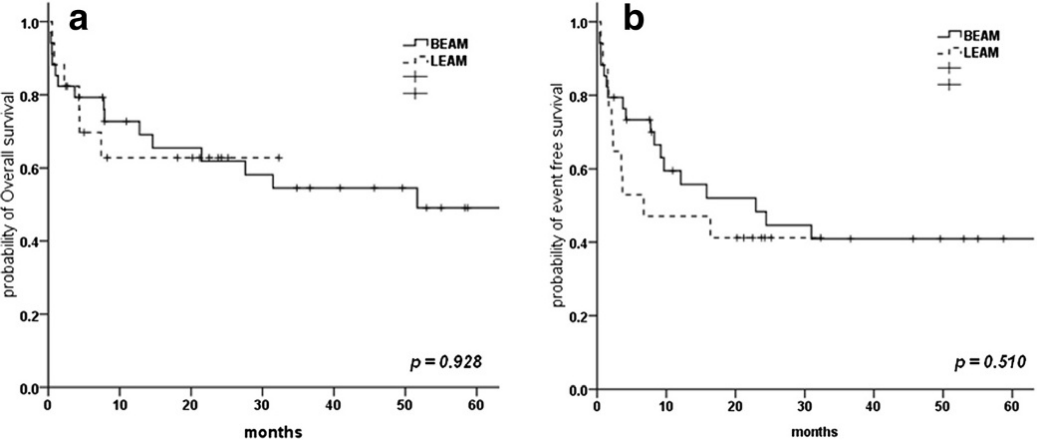
**Kaplan Meier survival estimate for (a) Overall survival and (b) Event free survival for patients treated with BEAM or LEAM.**

At a median follow up of 36.6 months (range, 0.26 – 130.9 months), the median OS for the whole cohort was 51.6 months and median EFS was 16.3 months. The estimated 5 year OS and EFS for the entire study group was 49.3% (SE 0.08) and 39.6% (SE 0.07) respectively. No significant difference was noted in the estimated 2 year OS [BEAM - 61.7% (SE 0.08) vs. LEAM 62.7% (SE 0.12), p = 0.928] and EFS [BEAM – 44.6% (SE 0.09) vs. LEAM – 41.1% (SE 0.11), p = 0.510)] between the two HDC study groups (Figure 1).

## Discussion

High dose chemotherapy and ASCT for lymphomas utilizes several drug combinations and in the absence of randomized comparative data, BEAM is the regimen most widely used because of its apparent tolerability and established efficacy. Shortage of old drugs is a worldwide problem, and while alternative regimens do follow the principles of combination chemotherapy, this requires attention and action by our health authorities. BCNU needs an alcoholic solvent for intravenous infusion and lately its availability world over was stalled by technical problems in its production (Leslie 2009). CCNU (lomustine) another nitrosurea, available for oral administration seemed the most logical substitute, and the modified regimen (LEAM) use was reported by some authors (Perfetti et al. 2009; Ramzi et al. 2012). Moreover, investigators have used other lomustine based regimens for high dose chemotherapy conditioning for lymphomas; partly in view of the concern for higher rates of pulmonary toxicity associated with BCNU compared to CCNU. LACE (lomustine, cytarabine (Ara-C), cyclophosphamide, etoposide) (Perz et al. 2007) and CCNU substitution for BCNU in the CBV (cyclophosphamide, BCNU, etoposide) regimen (Stuart et al. 2001), are some other lomustine based regimens that has shown comparable results. However, randomized studies are absent and direct comparative studies are lacking. In this single centre study, we examined the toxicities and efficacy of the recently used LEAM regimen and compared it with standard BEAM.

We found no significant difference in the global measures of peri-transplant outcomes in terms of engraftment, febrile complications, regimen related organ toxicities, intravenous antibiotic use, duration of hospitalization and 100 days TRM between the two high dose regimens. Also there was no difference in the short term survival and relapse/progression among the two groups.

Time to both neutrophil and platelet engraftments were not significantly different in the two treatment schedules and were comparable to that reported in the literature for BCNU and CCNU based regimens (Perz et al. 2007; Puig et al. 2006). Similarly, documented infections were not different between the groups. Gastrointestinal toxicity was the commonest grade 3/4 toxicity with mucositis (67%) and diarrhea (45%) evenly distributed in both the groups. Similar rates for mucositis have been reported for BCNU based regimens (Mills et al. 1995; Wang et al. 2004). Although BCNU is reported to have more pulmonary toxicity compared to CCNU (Cordonnier et al. 1983; Reece et al. 1991), no significant difference was seen in the occurrence of grade 3 or 4 pulmonary toxicity between the study groups (9% and 6%) in the present cohort. In the study by Stuart et al. utilizing a CCNU based regimen for relapsed HL (Stuart et al. 2001), incidence of interstitial pneumonitis with high dose CCNU exceeded prior studies with BCNU, 63% incidence of interstitial pneumonitis (IP) compared to 25% to 28% reported for BCNU at dose ≥ 600 mg/m^2^ (Schmitz et al. 1993; Wheeler et al. 1990). The authors attributed this to the use of a broader definition for IP, inclusion of patients with preexisting IP and prior exposure to radiotherapy. Notably, despite the fact that there were significantly more number of patients with chemorefractory disease and high risk co morbidity index in the LEAM subgroup, only 1 (6%) patient had a high risk (≥ 9) cumulative toxicity score compared to 7 (20%) in the BEAM subgroup though the difference was not significant.

In our study, 100 day TRM of 15.6% for the whole cohort was a little higher than the currently reported figures globally (Sureda et al. 2001; Wang et al. 2004) however, the difference between the two groups was not significant. The early survivals results for OS and EFS (estimated 2 years – 61.7% and 44.6% for BEAM and 62.7% and 41.1% for LEAM respectively) at a median follow up of 36.6 months are also similar for the two groups. In the report on LEAM by Perfetti et al., at a median follow-up of 3.66 years OS was 72% and disease free survival (DFS) was 66% (Perfetti, et al. 2009). In another study of a similar regimen CEAM, with a median follow-up of 27 months, median DFS was 20 months and median OS was not reached (Ramzi et al. 2012). Equivalent results for patients in the LEAM subgroup in our present study, even with more chemorefractory disease and high co morbidity index warrants further confirmation in a randomized study with larger number of patients. If this is confirmed it is possible that LEAM may be superior in chemosensitive disease.

With this study we document the initial comparative results in a small group of patients and conclude that the two regimens, BEAM and LEAM are equivalent for early peri-transplant outcomes and survival. And, lomustine can be a cheaper and readily available alternative to BCNU.
